# Risk assessment and predicting outcomes in patients with depressive symptoms: a review of potential role of peripheral blood based biomarkers

**DOI:** 10.3389/fnhum.2015.00018

**Published:** 2015-02-02

**Authors:** Bhautesh D. Jani, Gary McLean, Barbara I. Nicholl, Sarah J. E. Barry, Naveed Sattar, Frances S. Mair, Jonathan Cavanagh

**Affiliations:** ^1^General Practice and Primary Care, Institute of Health and Wellbeing, College of Medical, Veterinary and Life Sciences, University of GlasgowGlasgow, UK; ^2^Robertson Centre for Biostatistics, Institute of Health and Well Being, College of Medical, Veterinary and Life Sciences, University of GlasgowGlasgow, UK; ^3^BHF Glasgow Cardiovascular Research Centre, Institute of Cardiovascular and Medical Sciences, College of Medical, Veterinary and Life Sciences, University of GlasgowGlasgow, UK; ^4^Mental Health and Wellbeing, Institute of Health and Wellbeing, College of Medical, Veterinary and Life Sciences, University of GlasgowGlasgow, UK

**Keywords:** peripheral biomarkers, depression, treatment response, risk assessment, outcomes

## Abstract

Depression is one of the major global health challenges and a leading contributor of health related disability and costs. Depression is a heterogeneous disorder and current methods for assessing its severity in clinical practice rely on symptom count, however this approach is unreliable and inconsistent. The clinical evaluation of depressive symptoms is particularly challenging in primary care, where the majority of patients with depression are managed, due to the presence of co-morbidities. Current methods for risk assessment of depression do not accurately predict treatment response or clinical outcomes. Several biological pathways have been implicated in the pathophysiology of depression; however, accurate and predictive biomarkers remain elusive. We conducted a systematic review of the published evidence supporting the use of peripheral biomarkers to predict outcomes in depression, using Medline and Embase. Peripheral biomarkers in depression were found to be statistically significant predictors of mental health outcomes such as treatment response, poor outcome and symptom remission; and physical health outcomes such as increased incidence of cardiovascular events and deaths, and all-cause mortality. However, the available evidence has multiple methodological limitations which must be overcome to make any real clinical progress. Despite extensive research on the relationship of depression with peripheral biomarkers, its translational application in practice remains uncertain. In future, peripheral biomarkers identified with novel techniques and combining multiple biomarkers may have a potential role in depression risk assessment but further research is needed in this area.

## Introduction

### Heterogeneity in depressive symptoms

Depression is a heterogeneous disorder with a spectrum ranging from minor/sub threshold to major depressive disorder (MDD) (Rodriguez et al., 2012). According to the latest global disease burden study, depressive disorders (MDD and sub threshold/minor depression) are the leading cause of disability and disease burden globally (Ferrari et al., 2013). The methods currently available for risk assessment and stratification of symptom severity for patients presenting with depressive symptoms rely predominantly on counting the absolute number of depressive symptoms present but there is no universally accepted standardized definition. The Diagnostics and Statistical Manual (DSM)-IV's diagnosis of a MDD requires the presence of at least 5 out of 9 symptoms of depression with significant impairment or distress, while those presenting with at least 2 but less than 5 symptoms and no previous history of MDD are stratified as sub threshold or minor depression (American Psychiatric Association, 2000). The category of sub threshold depression has been removed from recently published DSM-V (American Psychiatric Association, 2013). On the other hand, the International Classification of Diseases (ICD-10) stratifies depressive symptoms on the basis of the number of depressive symptoms present into mild (4 out of 10), moderate (5 or 6 out of 10) and severe (7 or more out of 10) depressive episode (WHO, 2010). However, this approach has been questioned owing to lack of consensus (Wittchen et al., 2001; Hegerl et al., 2012) and because it ignores the complexity and diversity of depressive symptoms (Goldberg, 2011). The bulk of patients reporting with depressive symptoms are managed in primary care; however the rate of accurate stratification of depressive symptoms in primary care was less than 50% based on a meta-analysis involving more than 50,000 patients (Mitchell et al., 2009). Minor or sub threshold depression has been associated with severe deficits in psychological well-being and quality of life, progression to major disorder and increased mortality (Cuijpers and Smit, 2002; Lyness et al., 2006; Nierenberg et al., 2010), underlining the need for its early identification and appropriate treatment.

### Management of depression

The uncertainty in stratifying depression severity based on symptom count affects subsequent management. A review of treatment guidelines for depression across North America and Europe revealed that “mild MDD and sub threshold depression has the most variance in recommendations”; with suggested approaches ranging from watchful waiting to active treatment with antidepressants (Davidson, 2010). In the last decade, three separate meta-analyses reported that the efficacy of antidepressants is related to the initial severity of depression and they may not be effective in the treatment of mild depression (Khan et al., 2002; Kirsch et al., 2008; Fournier et al., 2010). However, this view has been challenged recently with emerging evidence suggesting that the efficacy of antidepressants in depression may not be related to its initial severity (Gibbons et al., 2012; Fountoulakis et al., 2013). Psychological therapies have been found to be effective in the management of mild depression but they have not been subjected to the same level of scrutiny as pharmacological therapies as yet (Cuijpers et al., 2007). The ambiguity surrounding stratifying the severity of depression based on symptom count and its subsequent management could partially explain why most patients with depression do not receive adequate treatment and many treated patients develop treatment resistance and relapse (Thase, 2006; Nemeroff, 2007). Therefore, different approaches for risk assessment and severity stratification of patients presenting with depressive symptoms are urgently required.

### Pathogenesis of depression

The etiopathogenesis of depression has been extensively studied over the last five decades with various explanatory mechanisms involving different physiological systems, suggesting heterogeneity (Zunszain et al., 2011). The “monoamine hypothesis of depression” was proposed in the 1960s with early work showing increased levels of plasma tryptophan (serotonin precursor) in patients with major depression (Coppen et al., 1973). Failure to suppress cortisol in response to dexamethasone in patients with depression was the initial finding which supported the role of hypothalamic-pituitary-adrenal (HPA) axis hyperactivity in the pathophysiology of depression (Carroll et al., 1981). The “cytokine hypothesis” suggests that depression is triggered, in part, via inflammatory processes in response to various internal and external stressors, following some seminal work in the early 1990s (Maes et al., 1991). This hypothesis has been further developed to suggest that inflammatory, oxidative and nitrosative stress are causally related to depression and increased translocation of lipopolysaccharide from gram negative bacteria may aggravate these pathways (Maes, 2008). The “neurogenesis hypothesis” of depression proposes that depression is characterized by neurodegeneration and impaired neurogenesis in the brain, in particular the hippocampus region (Sapolsky, 2004). The bi-directional relationship between metabolic syndrome and depression and their common pathophysiological pathways has been reported extensively (McIntyre et al., 2009; Vancampfort et al., 2013). Of course, several hypotheses may overlap or be relevant here.

### Biomarkers of depression

A biomarker can be defined as a characteristic that is objectively measured and evaluated as an indicator of normal biologic processes, pathogenic processes, or pharmacologic responses to a therapeutic intervention (Biomarkers Definitions Working Group, 2001). The research into pathogenesis of depression has led to a strong evidence base supporting a cross-sectional relationship between depressive symptoms and a number of different biomarkers pertaining to some of the physiological systems described above, but their role, if any, in predicting clinical outcomes in depression remains unclear (Macaluso et al., 2012). Peripheral biomarkers (blood based) are relatively non-invasive (other than the need for a blood sample) and easier to measure; hence they have a greater potential for translational application into routine clinical practice, when compared to imaging, genetic and CNS biomarkers. Peripheral biomarkers such as those related to HPA axis, inflammatory and monoamine systems may have a role in the diagnosis of depression by identifying a “biological sub-type” of depression, and in prognostication of depression by predicting treatment response, which in turn could help in its severity stratification and management (Fisar and Raboch, 2008; Leuchter et al., 2010; Schmidt et al., 2011). Various inflammatory and oxidative stress biomarkers have been proposed to have a potential role, not only in predicting antidepressant response, but also in enhancing treatment matching and onset prediction in patients with depression (Lopresti et al., 2014). For example, in a study based on a multi-center trial involving depression patients, showed an interaction between antidepressants and C-reactive protein with patients with raised CRP more likely to respond to nortripytline than escitalopram (Uher et al., 2014).

### Aims of the review

To attempt to address this issue, we examine the evidence base exploring the potential role of peripheral biomarkers at baseline in predicting future outcomes in patients with depression. We discuss the potential role of peripheral biomarkers identified using novel and emerging techniques such as proteomics, metabolomics, genetics, and epigenetics in risk assessment and outcome prediction in patients with depressive symptoms. We also review the relationship between depressive symptoms and a composite index score derived using multiple peripheral biomarkers such as allostatic index (AI) and discuss its possible role in future, in management of depression.

## Methods

**Figure 1:**
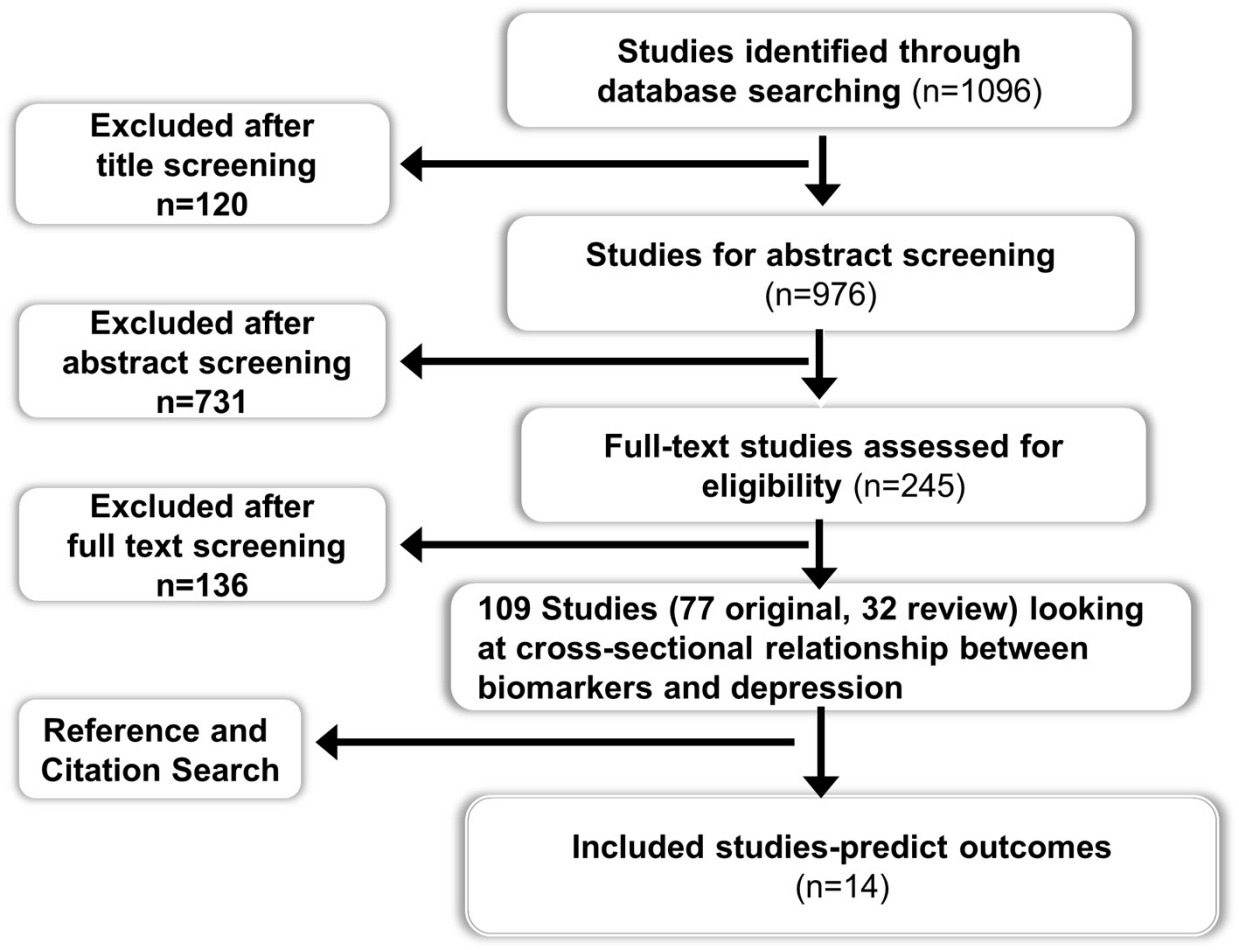
**Flow chart for the systematic review on the role of peripheral biomarkers predicting outcomes in patients with depression**.

Two electronic databases (Ovid Medline and Embase) were searched for studies published between 1946 and Jan 2013 using the MESH terms “Biological markers” AND “Depression.” All original and review studies using peripheral biomarkers at baseline as a risk assessment tool for predicting future outcomes in patients with depression were included. Clinical outcomes pertaining to both mental health (e.g., depressive symptoms) and physical health (e.g., cardiovascular event) were included. Only studies published in English language were considered for inclusion. Studies related to animal, imaging biomarkers, cerebrospinal fluid biomarkers, and mood disorders other than depression were excluded. Studies which investigated the role of depressive symptoms and peripheral biomarkers independently in predicting adverse physical outcomes but did not examine the interaction between depressive symptoms and peripheral biomarkers, or in other words did not perform a sub-group analysis in patients with depression, were excluded. Studies that investigated changes in peripheral biomarker levels following treatment for depression and which didn't report any correlation between baseline biomarker levels and depressive symptoms were excluded as the aim of this review was to focus on the use of peripheral biomarkers at baseline or pre-treatment as a predictive tool of clinical outcome (both mental and physical), rather than a change in biomarker level itself. The search strategy returned 1096 studies from two databases after excluding duplicates (see Figure 1 for details).

Title, abstract and full text screening followed by reference and citation searching and data extraction were carried out independently by two researchers (Bhautesh D. Jani and Gary McLean). The data extraction comprised of study sample size and country, type of study and setting, details of how depression was diagnosed and treated, follow-up duration, biomarkers assessed, clinical outcomes studied and potential bias in the results. The description of methodology used by included studies for biomarker measurement and the source of peripheral biomarker (i.e., serum or plasma or whole blood) was also reviewed in data extraction.

## Results

### Included studies and their characteristics

There was extensive evidence (109 studies) exploring and supporting the cross-sectional relationship between depression and different peripheral biomarkers. However, only a minority of studies (*n* = 14) explored the use of peripheral biomarkers to predict outcomes in patients with depression. Fifteen papers were included for data extraction; which consisted of nine prospective cohort studies (Duval et al., 1996; Perez et al., 1998; Alvarez et al., 1999; Johnston et al., 1999; Lanquillon et al., 2000; Ladwig et al., 2005; Binder et al., 2009; Jokinen and Nordstrom, 2009; Baune et al., 2012), three case-control studies (Arolt et al., 2003; Baldwin et al., 2006; Jang et al., 2008), two randomized controlled trials (Kin et al., 1997; Raison et al., 2013) and one meta-analysis (Ribeiro et al., 1993). Full details of included studies are summarized in Table 1.

**Table 1:** **Summary of included studies**.

	**Type of study and setting**	**Sample size at follow-up**	**Depression diagnosis criteria**	Source of Biomarker (Serum/Plasma/whole blood), Biomarkers Assessed (and type of biomarker) *implies statistically significant (*p*-value < 0.05)	**Treatment offered**	**Follow-up duration**	**Outcomes studied**
Alvarez et al., 1999; France	Cohort; psychiatry inpatients	*N* = 8	MADRS ≥ 20	Serum 5 plasma fluoxetine (Neurotransmitter metabolism)	Fluoxetine 20 mg	28 days	Treatment response defined as 50% reduction in MADRS scores from baseline
				Plasma norfluoxetine (Neurotransmitter metabolism)			
				Plasma fluoxetine plus norfluoxetine (Neurotransmitter metabolism)			
				Plasma 5 HT (Neurotransmitter metabolism)			
				Serum 5HT (Neurotransmitter metabolism)			
Arolt et al., 2003; Germany	Case-control; psychiatry inpatients	*N* = 25 (MDD), *N* = 25 (healthy controls)	Composite International	Plasma S100 B protein* (Neurotrophic)	Different groups of anti-depressants	28 days	Treatment Response defined as 50% reduction in HDRS from baseline
			Diagnostic Interview for DSM-IV criteria for MDD				
Baldwin et al., 2006; UK	Case-control; Community	*N* = 28 (MDD), *N* = 35 (healthy controls)	SCID for MDD	HDL cholesterol* (Metabolic)	Not specified	3.1 years	Poor outcome of depression based on author described criteria assessed by SCID
				LDL cholesterol (Metabolic)			
				BMI (Metabolic)			
				ESR (Inflammatory)			
				Pre-prandial glucose (Metabolic)			
				Source: Serum/Plasma/Whole blood was not specified			
Baune et al., 2012; Australia	Cohort; community	*N* = 73	GDS ≥ 6	Serum IL1β (Inflammatory)	Not specified	23.39 months (average)	Remission of depression symptoms defined as GDS <6
				Serum IL6 (Inflammatory)			
				Serum IL8 * (Inflammatory)			
				Serum IL10 (Inflammatory)			
				Serum IL 12p70 * (Inflammatory)			
				sVCAM-1 (Inflammatory)			
				Serum PAI-1 (Inflammatory)			
				SAA (Inflammatory)			
				Serum TNF-α (Inflammatory)			
				Serum CRP (Inflammatory)			
Duval et al., 1996; France	Cohort study; psychiatry Inpatients	*N* = 30	Unstructured interview for DSM-IV for MDD	Plasma TSH (Neuroendocrine)	1.Amitriptyline (*n* = 13)	1 month	1. Remission of depression symptoms defined as HDRS<8
				Plasma Free T3 (Neuroendocrine)	2.Fluoxetine (*n* = 9)		2. “Partial Response” (treatment response) defined as HDRS 8–15
				Plasma Free T4 (Neuroendocrine)	3.Toloxatone (*n* = 8)		
				Plasma TSH response to Protirelin stimulation* **(*for outcomes 1 and 2*)** (Neuroendocrine)			
				Plasma Free T3 response to Protirelin stimulation (Neuroendocrine)			
				Plasma Free T4 response to Protirelin stimulation (Neuroendocrine)			
Jang et al., 2008; South Korea	Case-control; Psychiatry Outpatients	*N* = 59 (MDD), *N* = 34 (healthy controls)	SCID for MDD	Serum S100B protein * (Neurotrophic)	Different groups of anti-depressants	6 weeks	Treatment response defined as 50% reduction in HDRS from baseline
Johnston et al., 1999; UK	Cohort; Psychiatry Outpatients and Inpatients	*N* = 34	SCID for MDD	Plasma Norepinephrine* (Neuroendocrine)	Not specified	8 years (average)	Poor Outcome defined by Depression Outcome Scale and Lee and Murray criteria
				Plasma Cortisol (Neuroendocrine)			
Jokinen and Nordstrom, 2009; Sweeden	Cohort; Psychiatry Inpatients	*N* = 346	DSM- IV criteria for all mood disorders, diagnostic method unspecified	Plasma Cortisol* **(for outcomes 1 and 2)** (Neuroendocrine)	Not specified	18 years (average)	1. Death due to natural causes
				Plasma Dexamethasone non-suppression* **(for outcomes 1 and 2)** (Neuroendocrine)			2. Cardiovascular deaths
Kin et al., 1997; Multi-center	RCT with 3 arms; not specified	*N* = 70 randomized into 3 arms	HDRS ≥ 18	Plasma dexamethasone non-suppression* **(only in Nortriptyline arm)** (Neuroendocrine)	3 arms: 1. Nortriptyline 75 mg	7 weeks	Treatment Response defined as 50% reduction in HDRS from baseline
					2.Moclobemide 400 mg		
					3. Placebo		
Ladwig et al., 2005; Germany	Cohort; Community	*N* = 975 (only males)	von Zerssen affective symptom check list with a score ≥11	Serum Highly sensitive CRP high risk group > 3 mg/ml* (Inflammatory)	Not specified	7.7 years (average)	1. Myocardial Infarction 2. Sudden cardiac death
Lanquillon et al., 2000; Germany	Cohort; Psychiatry inpatients	*N* = 24	SCID for MDD	Whole blood Lymphocyte count (Inflammatory)	Amitriptyline in increasing dose	6 weeks	Treatment Response defined as 50% reduction in HDRS and MADRS from baseline
				Whole blood Monocyte count (Inflammatory)			
				Whole blood Ratio lymphocyte/monocyte* (Inflammatory)			
				Whole blood CRP (Inflammatory)			
				Whole blood ESR (Inflammatory)			
				Whole blood IL-6 * (Inflammatory)			
				Whole blood TNF-alpha (Inflammatory)			
Perez et al., 1998; Spain	Cohort; Psychiatry Inpatients	*N* = 83	HDRS ≥ 17	Plasma 5HIAA (Neurotransmitter)	Different groups of anti-depressants	6 weeks	Treatment Response defined as 50% reduction in HDRS from baseline
				Plasma Total Tryptophan (Neurotransmitter)			
				Plasma 5 HT (Neurotransmitter)			
				Platelet 5 HT with high concentration 800 ng/10^9^ platelets* **(stronger relationship in SSRI sub-group)** (Neurotransmitter)			
Raison et al., 2013; US	RCT with 2 arms; Community	*N* = 60 randomized into two arms	Treatment resistance Depression diagnosed using Massachusetts	Plasma Highly sensitive CRP high risk group > 5 mg/ml (Inflammatory)	2 arms: 1.Infliximab infusions × 3 2. Placebo	12 weeks	1. Treatment Response defined as 50% reduction in HDRS from baseline
			General Hospital Staging method for treatment resistance ≥ 2				2. Remission of depression symptoms defined as HDRS<8
Ribeiro et al., 1993; US	Meta-analysis with 3 different research questions (RQ1-3)	RQ-1	Heterogeneous, including different symptoms scores and interview techniques	Dexamethasone non-suppression* **(for RQ2 only)** Source: Serum/Plasma/Whole blood was not specified for the included studies	Various	Not specified	1. (RQ1) “Treatment Response”
		*N* = 1273					
		RQ-2	Heterogeneous, including different symptoms scores and interview techniques		Various	1–7 weeks	1. (RQ1) “Treatment Response”
		*N* = 412					
		RQ-3	Heterogeneous, including different symptoms scores and interview techniques		Various	1–60 months	3. Long term outcome of depression based on predefined author criteria
		*N* = 411					

*MADRS, Montgomery Asperg Depression Rating Scale; 5HT, 5 Hydroxytryptamine; HDRS, Hamilton Depression Rating Scale; DSM-IV, Diagnostic and Statistical Manual IV; MDD, Major Depressive Disorder; SCID, Structured Diagnostic Interview for DSM-IV; HDL, High Density Lipoprotein; LDL, Low Density Lipoprotein; ESR, Erythrocyte Sedimentation Rate; BMI, Body Mass Index; GDS, Geriatric Depression Scale; IL, Interleukin; sVCAM-1, Serum Vascular Cell AdhesionMolecule -1; PAI-1, Plasminogen Activator Inhibitor-1; SAA, Serum Amyloid A; TNF-α, Tumor, Necrotic Factor-alpha; CRP, C Reactive Protein; TSH, Thyroid Stimulating Hormone; RCT, Randomized Controlled Trial; 5-HIAA, 5-Hydroxyindoleacetic acid, RQ, Research Question*.

Sample sizes ranged from 8 to 986 with sample sizes of less than 50 participants in00206 studies (Duval et al., 1996; Alvarez et al., 1999; Johnston et al., 1999; Lanquillon et al., 2000; Arolt et al., 2003; Baldwin et al., 2006), while three studies had a sample size of 25 or less (Alvarez et al., 1999; Lanquillon et al., 2000; Arolt et al., 2003). Follow-up duration ranged from 4 weeks to 18 years with the follow-up duration being less than 6 months in 9 studies(Duval et al., 1996; Kin et al., 1997; Perez et al., 1998; Alvarez et al., 1999; Lanquillon et al., 2000; Arolt et al., 2003; Jang et al., 2008; Binder et al., 2009; Raison et al., 2013) while only 5 studies followed their subjects for more than 12 months (Johnston et al., 1999; Ladwig et al., 2005; Baldwin et al., 2006; Jokinen and Nordstrom, 2009; Baune et al., 2012). Six studies used a diagnostic interview technique (Duval et al., 1996; Johnston et al., 1999; Lanquillon et al., 2000; Arolt et al., 2003; Baldwin et al., 2006; Jang et al., 2008) and seven studies used a depression rating scale (Kin et al., 1997; Perez et al., 1998; Alvarez et al., 1999; Ladwig et al., 2005; Binder et al., 2009; Baune et al., 2012; Raison et al., 2013) while diagnostic method was not specified in one of the included studies (Jokinen and Nordstrom, 2009). The nature of the treatment was specified in nine studies(Duval et al., 1996; Kin et al., 1997; Perez et al., 1998; Alvarez et al., 1999; Lanquillon et al., 2000; Arolt et al., 2003; Jang et al., 2008; Binder et al., 2009; Raison et al., 2013); the relationship between outcome and baseline depression severity was only taken into account in 5 studies (Duval et al., 1996; Johnston et al., 1999; Lanquillon et al., 2000; Arolt et al., 2003; Baldwin et al., 2006). The included meta-analysis had a variable sample size and follow-up duration depending on the different research questions considered by the study and the diagnostic methods used were heterogeneous including various symptoms scores and interview techniques (Ribeiro et al., 1993).

### Biomarkers studied and method of collection

**Figure 2:**
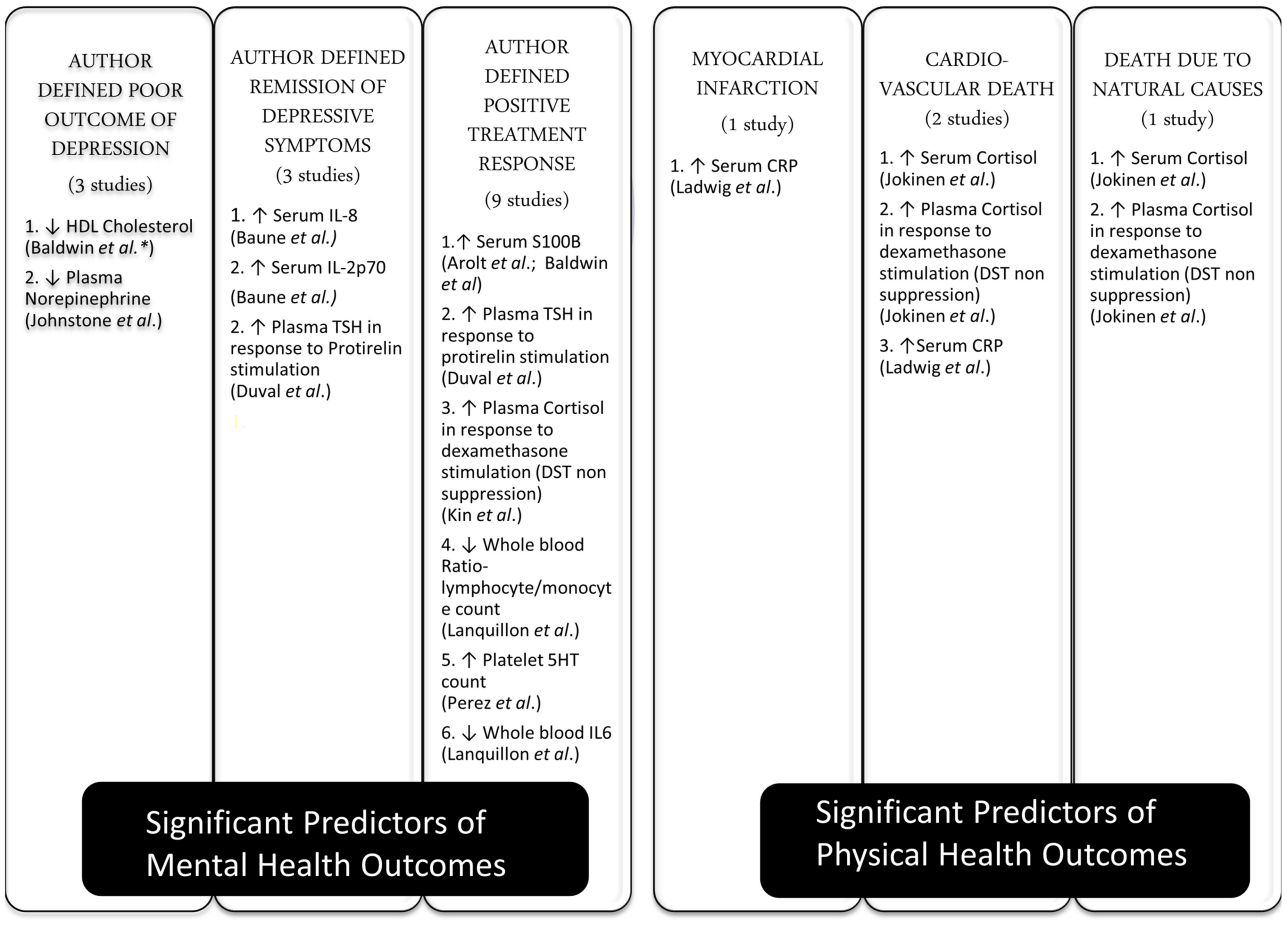
**Different outcomes in depression and their significant predictors**. This Figure describes the various mental and physical health outcomes considered by included studies in the review, the number of studies which examined each outcome, the peripheral biomarkers which were found to have a statistically significant impact in predicting each outcome and the direction of the relationship. DST, Dexamethasone Suppression Test; CRP, C Reactive Protein; IL, Interleukin; 5HT, 5 Hydroxytryptamine; TSH, Thyroid Stimulating Hormone; ↑: higher, ↓: lower. ^*^The study did not specify the source of the biomarker studied (i.e., serum or plasma).

The included studies assessed 36 different peripheral biomarkers at baseline as a predictor of clinical outcomes. These biomarkers were measured in serum or plasma and could be broadly classified as pertaining to inflammatory (*n* = 14), neurotransmitter metabolism (*n* = 9), neuroendocrine (*n* = 8), metabolic (*n* = 4), and neurotrophic (*n* = 1) systems. All included studies assessed statistical significance based on the criteria of having a *p*-value less than 0.05. Twelve biomarkers were found to be statistically significant in predicting outcomes (summarized in Figure 2). Inflammatory (Lanquillon et al., 2000; Ladwig et al., 2005; Baldwin et al., 2006; Baune et al., 2012; Raison et al., 2013) and neuroendocrine (Ribeiro et al., 1993; Duval et al., 1996; Kin et al., 1997; Johnston et al., 1999; Jokinen and Nordstrom, 2009) biomarkers were each assessed in five of the included studies, followed by neurotransmitter (Perez et al., 1998; Alvarez et al., 1999; Johnston et al., 1999) biomarkers in three studies, neurotrophic (Arolt et al., 2003; Jang et al., 2008) biomarker in two studies, while metabolic (Baldwin et al., 2006) biomarkers were assessed in only one study.

The source of peripheral biomarker measurement was plasma in half of the included studies (*n* = 7) (Duval et al., 1996; Kin et al., 1997; Perez et al., 1998; Johnston et al., 1999; Arolt et al., 2003; Jokinen and Nordstrom, 2009; Raison et al., 2013); serum in three studies (Ladwig et al., 2005; Jang et al., 2008; Baune et al., 2012); whole blood (Lanquillon et al., 2000) and mixed (both serum and plasma) (Alvarez et al., 1999) in 1 study each; and not reported in two of the included studies (Ribeiro et al., 1993; Baldwin et al., 2006). Four of the included studies did not describe the procedures of measuring peripheral biomarker in detail (Ribeiro et al., 1993; Duval et al., 1996; Baldwin et al., 2006; Jokinen and Nordstrom, 2009). Four of the included studies describe the anticoagulant used for collecting plasma samples with ethylenediaminetetraacetic acid (EDTA) used by two studies (Perez et al., 1998; Raison et al., 2013); and heparin (Arolt et al., 2003) and sodium citrate (Alvarez et al., 1999) used by one study each.

### Types of clinical outcomes studied and statistical methods

The majority of included studies (*n* = 12) (Ribeiro et al., 1993; Duval et al., 1996; Kin et al., 1997; Perez et al., 1998; Alvarez et al., 1999; Johnston et al., 1999; Lanquillon et al., 2000; Arolt et al., 2003; Baldwin et al., 2006; Jang et al., 2008; Baune et al., 2012; Raison et al., 2013) examined outcomes pertaining to mental health or depressive symptoms, with only two studies assessing physical health outcomes (Ladwig et al., 2005; Jokinen and Nordstrom, 2009). Author defined positive treatment response to anti-depressants with improvement in depressive symptoms [e.g., 50% reduction in depression rating scale Hamilton Depression Rating Scale (HDRS) from baseline] was the commonest outcome considered by nine included studies (Ribeiro et al., 1993; Duval et al., 1996; Kin et al., 1997; Perez et al., 1998; Alvarez et al., 1999; Lanquillon et al., 2000; Arolt et al., 2003; Jang et al., 2008; Raison et al., 2013). This was followed by other mental health outcomes such as author defined criteria for poor outcome of depressive symptoms (*n* = 3) (Ribeiro et al., 1993; Johnston et al., 1999; Baldwin et al., 2006) for e.g., Lee and Murray operational criteria for outcome in depression; and remission of depression symptoms (*n* = 3) (Duval et al., 1996; Baune et al., 2012; Raison et al., 2013) for e.g., HDRS <8 at follow-up. The physical health outcomes measured were cardiovascular deaths (*n* = 2) (Ladwig et al., 2005; Jokinen and Nordstrom, 2009), myocardial infarction (*n* = 1) (Ladwig et al., 2005) and death due to natural causes (*n* = 1) (Jokinen and Nordstrom, 2009). The usefulness of statistical models for physical outcomes was not compared against routinely used and evidence backed risk scores such as the Framingham score for cardiovascular events. Biomarkers were shown to be statistically significant in predicting all of the six outcomes considered, including mental and physical outcomes. Figure 2 summarizes the six different mental and physical health outcomes studied, the number of studies which examined each outcome, the 12 peripheral biomarkers which were noted to be statistically significant in predicting each outcome and the direction of the relationship between the biomarker and the outcome.

The Area Under Curve (AUC) statistic was presented only by 1 study, with AUC statistic for Dexamethasone suppression test (DST) reported as 0.65 for predicting increased incidence of cardiovascular deaths only for the male subset of their sample (Jokinen and Nordstrom, 2009). DST was found to have a significant impact in predicting three different outcomes in two different studies; which included adverse outcomes such as increased incidence of all-cause mortality and cardiovascular deaths (Jokinen and Nordstrom, 2009), and favorable outcome such as positive treatment response to anti-depressants (Kin et al., 1997). In the included meta-analysis, DST failed to have a significant impact in predicting positive response to anti-depressants but was significant in predicting positive response to placebo (Ribeiro et al., 1993). Elevated levels of serum S100B was the only biomarker which was found to have a statistically significant role in predicting the same clinical outcome (positive treatment response to anti-depressants) in more than one included studies (Arolt et al., 2003; Jang et al., 2008).

### Patient demographics and attrition rate

Description of patient demographics, co-morbid conditions and attrition at follow-up for included studies is provided in Table 2. Details of the age of participants were not described by three studies (Ribeiro et al., 1993; Kin et al., 1997; Baune et al., 2012); while information on gender distribution was missing from five studies (Ribeiro et al., 1993; Kin et al., 1997; Arolt et al., 2003; Baldwin et al., 2006; Baune et al., 2012). The socio-economic status of participants was very poorly described with only two studies (Ladwig et al., 2005; Raison et al., 2013) characterizing it and only one study (Ladwig et al., 2005) including socio-economic status in their statistical analysis. Patients with pre-existing chronic disease were excluded by the majority of the included studies (*n* = 8) (Duval et al., 1996; Perez et al., 1998; Johnston et al., 1999; Lanquillon et al., 2000; Arolt et al., 2003; Ladwig et al., 2005; Jang et al., 2008; Jokinen and Nordstrom, 2009) and chronic disease status was not considered or described by four of the included studies (Ribeiro et al., 1993; Kin et al., 1997; Alvarez et al., 1999; Baldwin et al., 2006). Of the two studies which included patients with co-existing chronic disease (Baune et al., 2012; Raison et al., 2013), only one study accounted for the number of co-morbidities in their statistical analysis (Raison et al., 2013). The reported participant attrition rate at follow-up varied from 0 to 44%; with two included studies (Ribeiro et al., 1993; Baune et al., 2012) not specifying the details of attrition.

**Table 2:** **Patient population and attrition rates in included studies**.

**Study**	**Mean Age in years (Standard Deviation, if available) and Sex F, Females; M, Males**	**Socio-economic status**	**Co-morbid medical conditions**	**Participant numbers Number of participants at baseline (B) and follow-up(FU); attrition in percentage**
Alvarez et al., 1999	45 (13.8)	Not described	Not described	10 B
	6F, 2M			8 FU 20% attrition
Arolt et al., 2003	46.4 (9.8)	Not described	Patients with co-morbid conditions excluded from study	25 B
	Not described			25 FU No attrition
Baldwin et al., 2006	73.9	Not described	Not described	50 B
	Not described			28 FU 44% attrition
Baune et al., 2012	Not described	Not described	Presence/absence of a list of medical conditions noted and entered into statistical analysis	73 B
				Sample size at follow-up not specified
Duval et al., 1996	39.8 (12.9);	Not described	Patients with co-morbid conditions excluded from study	30 B
	19M, 11F			30 FU No attrition
Jang et al., 2008	60.3;	Not described	Patients with co-morbid conditions excluded from study	59 B
	43F, 16M			59 FU No attrition
Johnston et al., 1999	47;	Not described	Patients with co-morbid conditions excluded from study	47 B
	24F, 10M			34 FU 27.6% attrition
Jokinen and Nordstrom, 2009	52 (16.4);	Not described	Patients with co-morbid conditions excluded from study	382 B
	256F, 126M			346 FU 9.4% attrition
Kin et al., 1997	Not described	Not described	Not described	95 B
				70 FU 26.3% attrition
Ladwig et al., 2005	57.75 (7.8);	Education status described and entered into statistical analysis	Patients with co-morbid conditions excluded from study	986 B
	975M			975 FU 1.1% attrition
Lanquillon et al., 2000	53.5;	Not described	Patients with co-morbid conditions excluded from study	35 B
	15F, 9M			24 FU 30.5% attrition
Perez et al., 1998	M 45 (2.9), F 44.9 (2.0);	Not described	Patients with co-morbid conditions excluded from study	89 B
	59F, 24M			83 FU 6.7% attrition
Raison et al., 2013	42.5(8.2) placebo group, 44.3 (9.4) intervention group;	Education and employment status described but not entered into statistical analysis	Notable exclusions- previous history of cancer, history of unstable cardiovascular, endocrinologic, hematologic, hepatic, renal, or neurologic disease (determined by Physical examination and laboratory testing). Number of co-morbid medical conditions noted and entered into statistical analysis	60 B
	40F, 20M			60 FU No attrition
Ribeiro et al., 1993	Not described	Not described	Not described	Not described

## Discussion

### Summary of findings

Our review shows that blood based peripheral biomarkers were statistically significant in predicting six different clinical outcomes in participants with depression. Outcomes related to both mental health (depressive symptoms) and physical health were statistically associated with pre-treatment levels of peripheral biomarkers; however only two studies investigated outcomes related to physical health. Twelve different biomarkers related to five different biological systems (inflammatory, neuroendocrine, neurotransmitter metabolism, neurotrophic, and metabolic) were found to have a potential role in predicting outcomes of depression. Despite extensive research on the biomarkers of etiopathogenesis of depression, there is limited published research exploring its translational application in clinical practice. Furthermore, the research is of generally limited quality and lacks clinical utility.

The included studies have several methodological problems. The study sample size was small and follow-up duration was short in the majority of included studies. The majority of included studies used questionnaire scores using symptom count for diagnosing depression at baseline, while the gold standard interview technique for depression diagnosis was used only by a minority. Baseline severity of depressive symptoms assessed using symptom count is associated with higher rate of relapse in patients with depression (Ishak et al., 2013) but accounting for the baseline severity of depressive symptoms was only undertaken by a minority of studies. There is a strong evidence base suggesting that depression is two to three time more prevalent in patients with co-existing chronic disease as compared to the general population (Egede, 2007; Moussavi et al., 2007; Mitchell et al., 2013) but the effect of co-morbidity on clinical outcomes was examined by only two studies.

Importantly, the clinical implications of the observed statistical relationships in the included studies were not well explained. The c-statistic or area under receiver operating characteristic (ROC) curve (Cook, 2007), which is regarded as one of the standard methods for evaluating clinical discriminating power of a statistical model, was reported by only one study. The usefulness of statistical models for physical outcomes in the included study was not compared against robustly validated and routinely used risk scores such as the Framingham score for cardiovascular events (D'Agostino et al., 2008). Finally, some of the biomarkers included in this review are complicated to measure and likely to be expensive. The source and method of measurement for biomarkers in the included studies were heterogeneous and this may have an influence on assay levels of the biomarkers measured (Tort et al., 2003; Wong et al., 2008; Yu et al., 2011). The cost implications of doing these tests were not considered in detail in the included studies and this is likely to be a relevant factor when considering their potential use in routine clinical practice. There is some evidence that peripheral biomarkers may have a role in stratifying depression severity by means of predicting various physical and mental health outcomes in depression but further more robust research needs to be done in this area to address the shortcomings of the available evidence.

### Outcomes based approach in depression severity stratification

The use of prediction rules and biomarkers to inform clinical decision making is not a novel concept. It has been used in making management decisions in a wide variety of clinical scenarios such as patients presenting with high cholesterol, atrial fibrillation, chest pain, ankle injury, and intensive care (Reilly and Evans, 2006). In psychiatry, it has been proposed to use this principle for predicting inpatient violence (Abderhalden et al., 2006). Depression contributes to disease burden not only owing to reduction in quality of life and functional productivity, but also due to the increased risk of adverse physical outcomes such as hospitalization and mortality (Ferrari et al., 2013). There is strong evidence showing an association of depression (MDD and mild depression) with increased risk of adverse physical outcomes such as all-cause mortality, cardiovascular disease, hypertension, stroke, diabetes, alzeimer's disease, obesity, and cancer (Penninx et al., 2013). Physical adverse outcomes associated with depression attribute to a significant amount of morbidity and mortality (Ferrari et al., 2013; Penninx et al., 2013). Consequently, it is imperative that the risk of adverse physical outcomes associated with depression should be considered while taking decisions regarding depression severity stratification and subsequent management. Crucially, the clinical utility of biomarkers in predicting physical outcomes in depression, if any, should be compared and validated against some of the established and available risk scoring systems (e.g., Framingham, D'Agostino et al., 2008) for physical outcomes.

### Role of peripheral biomarkers in identifying depression subtypes

The use of peripheral biomarkers in identifying different subtypes of depression has been explored by other studies in the literature. A meta-analysis reviewing the association between HPA axis hyperactivity (Dexamethasone non-suppression) and depression suggested a dose-response relationship, with patients with mild depression showing higher HPA hyperactivity compared to controls but lower than that of patients with MDD (Stetler and Miller, 2011). Peripheral inflammatory markers such as Tumor necrotic factor (TNF)-α and IL (Interleukin)-6, serum neopterin have been shown to have association with melancholic subtypes of MDD (Maes et al., 2012; Dunjic-Kostic et al., 2013). A review of metabolic and neuroendocrine biomarkers (Body mass index BMI, waist-hip ratio, fasting glucose, serum adrenocorticotropic hormone ACTH) in pre-menopausal women with MDD supported their role in identifying three different subtypes of MDD- melancholic, atypical and undifferentiated (Cizza et al., 2012). This suggests that peripheral biomarkers may have a useful role in addressing some of the challenges posed by heterogeneity of depression, with a particular biomarker likely to have a more useful role in a specific subtype of depression. However, before any decisions are made, much better high quality research is needed.

### Novel biomarkers in depression

In recent years, novel techniques in proteomics, metabolomics, genetics, and epigenetics have led to several new biomarkers being proposed in depression. Proteomic techniques have been used to identify nine differentiating proteins belonging to lipid metabolism and immune system from treatment naïve patients with depression, when compared against healthy controls (Xu et al., 2012). Similarly, metabolomic techniques such as nuclear magnetic response (NMR) based analysis of both urine and plasma have been utilized to identify differentiating proteins related to lipid metabolism and neurotransmitter system with good accuracy in treatment naïve patients with depression, when compared to healthy controls (Zheng et al., 2012a,b). The role of brain-derived neurotrophic gene polymorphisms, glucocorticoid receptor polymorphism and serotonin gene receptor have been studied in diagnosis and prognostification of depression with some encouraging results (Chi et al., 2010; Szczepankiewicz et al., 2011; Uher et al., 2011). Although the findings from genome wide association studies (GWAS) till date in depression have failed to make a major breakthrough, they may have a potential role in stratification of depression and further research is ongoing (Wray et al., 2012; Flint and Kendler, 2014). Thus, these emerging techniques and biomarkers may have a role in diagnosis, identifying specific subtypes of depression and prognostification in depression (Schneider and Prvulovic, 2013).

### Multiple biomarkers, allostatic load, and depression

The term allostasis refers to the adaptive physiological responses organisms activate when homeostasis is disrupted during acute stress, real or interpreted threats (McEwen and Stellar, 1993). When chronically activated, allostatic mechanisms become physiologically taxing—or an allostatic load (AL)—that consequently increase one's susceptibility to disease (McEwen, 1998). There is some early evidence to suggest that an index comprising multiple biomarkers or AI may exhibit a stronger relationship with depressive symptoms, especially in elderly populations, when compared with examination of individual biomarkers in isolation (Juster et al., 2011). The role of multiple biomarkers in risk assessment and predicting outcomes in patients with depression needs to be explored and compared against the role of individual biomarkers.

### Limitations

Our search strategy was limited to studies published in English language. A variety of other biomarkers such as genetic, imaging and CSF biomarkers may have a role in depression stratification by predicting clinical outcomes (Schneider and Prvulovic, 2013). However, this review considered only peripheral or blood-based biomarkers used in current clinical practice due to their comparative non-invasive nature and ease of measurement. The uncertainty surrounding management decisions in patients with depression in current practice is a particular issue at the time of initial presentation (Davidson, 2010). Hence, this review was focussed on addressing the issue of the use of peripheral biomarkers at baseline or pre-treatment as a predictive tool of clinical outcome (both mental and physical) and not on assessing changes in a peripheral biomarker level following treatment for depression.

### Future research

There is a need for further research in this area, involving large scale studies with longer duration of follow-up, better characterization of patient populations and inclusion of patients with chronic diseases. An “ideal” scientific process for a biomarker evaluation in clinical risk discrimination has been highlighted in other fields such as cardiovascular disease, a similar approach can be adopted for biomarkers of depression (Welsh et al., 2008). Further epidemiological studies of greater quality which minimize potential bias and evaluate clinical utility are urgently needed. Future studies also need to incorporate other physical health outcomes such as rate of cardiovascular events, incidence of cancer and all-cause mortality associated with depression and compare validity against established benchmarks, along with mental health outcomes related to depression symptoms.

## Conclusion

Pre-treatment levels of 12 different blood based peripheral biomarkers related to five different biological pathways were found to have a statistically significant relationship with outcomes in patients with depression. Six different outcomes in depression were predicted using these biomarkers, pertaining to both physical and mental health, but the clinical implications remain unclear. It appears likely that peripheral biomarkers may have an important role in helping clinicians to stratify depression severity and to predict clinical outcomes. However, the available evidence has multiple methodological limitations which must be overcome to make any real clinical headway; in particular, interaction between these biomarkers, depressive symptoms and co-morbid physical conditions needs to be explored further.

## Author contributions

Literature search was carried out by Bhautesh D. Jani and Barbara I. Nicholl. Title, abstract and full text screening followed by reference and citation searching and data extraction were carried out by Bhautesh D. Jani and Gary McLean. All authors contributed to the manuscript.

### Conflict of interest statement

The authors declare that the research was conducted in the absence of any commercial or financial relationships that could be construed as a potential conflict of interest.
